# Live Cell Imaging Unveils Multiple Domain Requirements for *In Vivo* Dimerization of the Glucocorticoid Receptor

**DOI:** 10.1371/journal.pbio.1001813

**Published:** 2014-03-18

**Authors:** Diego M. Presman, M. Florencia Ogara, Martín Stortz, Lautaro D. Alvarez, John R. Pooley, R. Louis Schiltz, Lars Grøntved, Thomas A. Johnson, Paul R. Mittelstadt, Jonathan D. Ashwell, Sundar Ganesan, Gerardo Burton, Valeria Levi, Gordon L. Hager, Adali Pecci

**Affiliations:** 1 Laboratory of Receptor Biology and Gene Expression, National Cancer Institute, NIH, Bethesda, Maryland, United States of America; 2 Department of Biological Chemistry, School of Sciences (FCEN), University of Buenos Aires (UBA), Buenos Aires, Argentina; 3 IFIBYNE-CONICET, School of Sciences (FCEN), University of Buenos Aires (UBA), Buenos Aires, Argentina; 4 Department of Organic Chemistry/UMYMFOR-CONICET, School of Sciences (FCEN), University of Buenos Aires (UBA), Buenos Aires, Argentina; 5 Henry Wellcome Laboratories for Integrative Neuroscience and Endocrinology, University of Bristol, Bristol, United Kingdom; 6 Laboratory of Immune Cell Biology, Center for Cancer Research, National Cancer Institute, Bethesda, Maryland, United States of America; 7 Biological Imaging Section, Research Technologies Branch, National Institute of Allergy and Infectious Diseases, NIH, Bethesda, Maryland, United States of America; University of California, San Francisco, United States of America

## Abstract

Glucocorticoids are essential for life, but are also implicated in disease pathogenesis and may produce unwanted effects when given in high doses. Glucocorticoid receptor (GR) transcriptional activity and clinical outcome have been linked to its oligomerization state. Although a point mutation within the GR DNA-binding domain (GRdim mutant) has been reported as crucial for receptor dimerization and DNA binding, this assumption has recently been challenged. Here we have analyzed the GR oligomerization state *in vivo* using the number and brightness assay. Our results suggest a complete, reversible, and DNA-independent ligand-induced model for GR dimerization. We demonstrate that the GRdim forms dimers *in vivo* whereas adding another mutation in the ligand-binding domain (I634A) severely compromises homodimer formation. Contrary to dogma, no correlation between the GR monomeric/dimeric state and transcriptional activity was observed. Finally, the state of dimerization affected DNA binding only to a subset of GR binding sites. These results have major implications on future searches for therapeutic glucocorticoids with reduced side effects.

## Abstract 2

The glucocorticoid receptor's oligomerization state is revealed to not correlate with its activity; this challenges the current prevailing view that this state defines its transcriptional activity.

## Author Summary

The powerful anti-inflammatory and immunosuppressive action of glucocorticoids have made them one of the most prescribed drugs worldwide. Unfortunately, acute or chronic treatment may have severe side-effects. Glucocorticoids bind to the glucocorticoid receptor (GR), a ligand-dependent transcription factor. GR regulates gene expression directly by binding to DNA or indirectly by modulating the activity of other transcription factors. It is currently accepted that the direct pathway is mostly responsible for glucocorticoids side-effects and that the oligomerization state of the GR (whether it is a dimer or a monomer) determines which pathway (direct or indirect) will prevail. Hence, scientists have tried to develop “dissociated ligands” able to specifically activate the GR indirect pathway. In the present work, we employed a novel microscopy method named the number and brightness assay, which measures GR oligomerization state inside the living cell. Our results suggest that—contrary to the established view—there is no clear correlation between the oligomerization state of GR and the mechanistic pathway the receptor will follow upon ligand binding. This discovery presents supporting evidence towards the increasing view of the inherent complexity of glucocorticoid action and might impact future approaches towards the design of safer synthetic glucocorticoids.

## Introduction

Glucocorticoids influence the activity of almost every cell in mammalian organisms, mainly through binding to the glucocorticoid receptor (GR). In the absence of ligand GR primarily localizes in the cytoplasm while the activated GR-ligand complex is mainly nuclear. Once in the nucleus, the GR regulates gene expression by directly binding to specific DNA sequences or by the interaction with, and modulation of other transcription factors [1]. These two main mechanisms of action were historically named GR transactivation and GR transrepression, respectively [2]. Even though GR homodimerization is considered an essential step in the GR-transactivation pathway, it is still not clear whether GR dimerizes before [3]–[6] or after [7]–[9] DNA binding; or which regions of the protein are functionally involved in the homodimerization process [10]. Nevertheless, as GR transactivation was originally correlated with side effects of long-term clinical use of glucocorticoids, intense efforts have been made to design GR ligands with “dissociated” glucocorticoid properties that exclusively activate the transrepression pathway [11]. Since the current model of the GR mechanism of action states that the monomeric/dimeric status of the receptor defines its transcriptional activity, most of the rational drug design strategies have been focused on the search for ligands that promote the monomeric (i.e., transrepression) form of GR [12].

GR is a modular protein organized into three major domains: the N-terminal ligand-independent activation function-1 domain; the central DNA-binding domain (DBD); and the C-terminal ligand-binding domain (LBD) [13]. Crystal structures of both DBD [14] and LBD [15] have been obtained separately but no reports have described a structure of the entire protein. The first crystal structure of the GR DBD revealed a dimerization region, and subsequent mutational studies partially defined a five amino acids sequence, named the D-loop, that could potentially be involved in GR dimer formation [8]. However, these earlier studies were performed with a GR fragment and entirely *in vitro*. Following this work, a point mutation within the human GR DBD (A458T) in the context of the entire protein was reported to be able to separate transactivation from transrepression and unable to dimerize [16], although no direct evidence supported the latter conclusion. The human GR^A458T^, mouse GR^A465T^, and rat GR^A477T^ have been commonly referred to as the “GRdim” mutants [17].

From a transcriptional standpoint, early studies characterized the GRdim mutant as unable to transactivate genes but able to transrepress both *in vitro* [16] and *in vivo* [18]. However, GRdim's inability to transactivate has been challenged after results that showed this mutant can induce gene expression in a sequence and context-dependent manner [19]–[21]. From a biophysical standpoint, the early GRdim studies established that dimerization was entirely dependent on the DBD region. However, a recent study confronted this idea by showing protein-protein interactions between GRdim molecules [22].

Here we performed *in vivo* mapping of the GR oligomerization state by using the number and brightness (N&B) method [23]. We present conclusive evidence showing dimerization of the GRdim mutant while an additional mutation in the LBD (I634A) severely compromises homodimer formation. Importantly, no correlation between oligomerization state, DNA binding, and transcriptional activity could be established. These results question a key paradigm in the quest for glucocorticoid “dissociated” ligands.

## Results

### Image Analysis Reveals GR Oligomerization State in Living Cells

**Figure 1:**
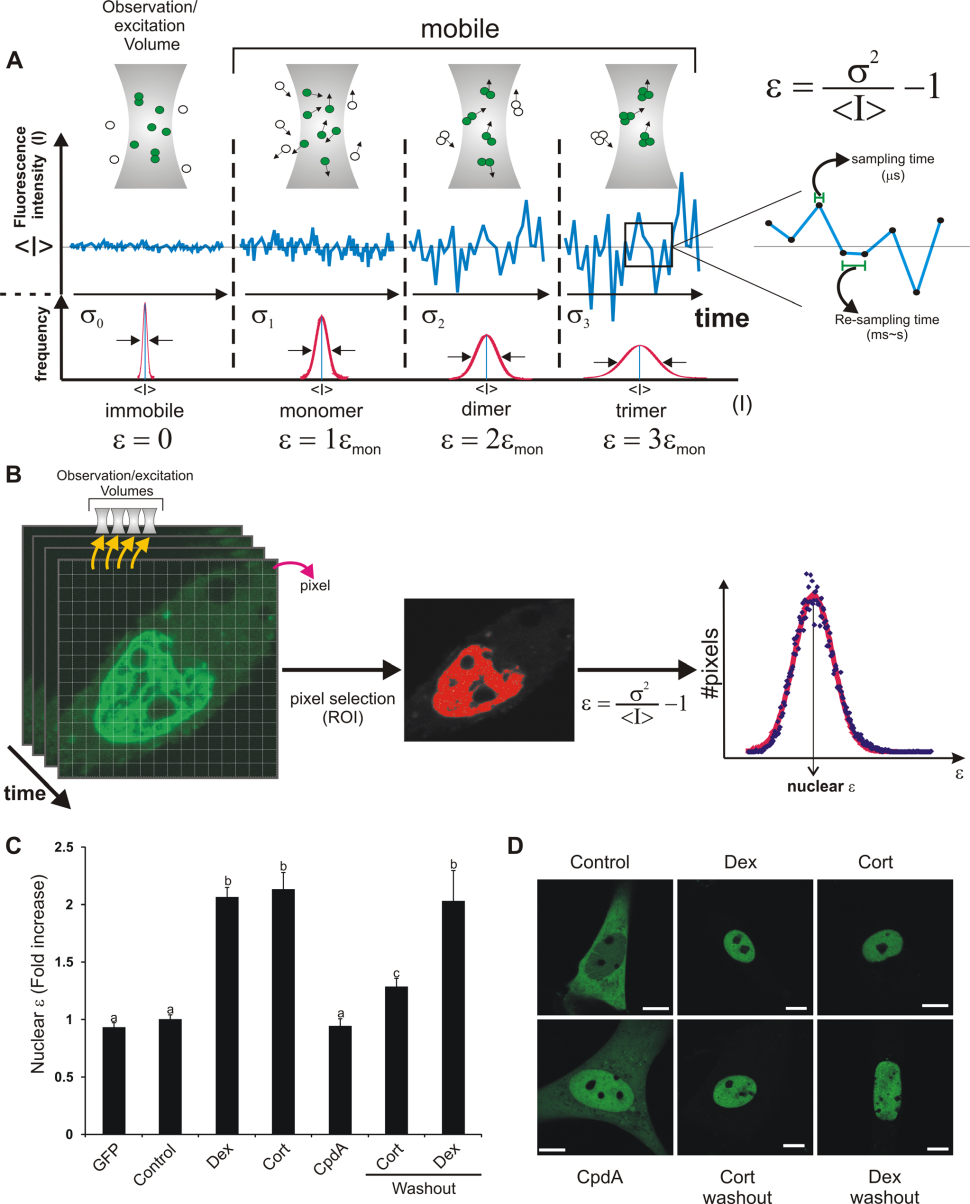
The N&B method and its application to GR dimerization. (A) The molecular brightness (ε) can be measured by analyzing the frame-to-frame intensity fluctuations in the confocal volume (∼fl). Fluctuations are analyzed by measuring the ratio between the variance of the intensity signal σ^2^ and the mean intensity value <I>. When fluorescent molecules are immobile, this ratio describes instrument noise which follows a Poisson distribution (σ^2^ = <I>). If the signal fluctuates due to mobile molecules, the ratio σ^2^/<I> is directly proportional to the ε of the diffusing species. The scheme illustrates the standard deviation for an equal number of fluorophores organized as immobile monomers (σ0), mobile monomers (σ1), dimers (σ2), or trimers (σ3). Figure adapted from Hellriegel and colleagues [55]. The sampling time (i.e., pixel dwell time) has to be short enough that the intensity fluctuations are not averaged out (∼µs) while the re-sampling time (i.e., frame time) must be longer (∼ms-s) to measure independent fluctuations due to different populations of molecules at the same pixel [23]. (B) To obtain ε of a fluorescent protein in living cells, a stack of images is acquired. Pixels that correspond to a specific region of interest (ROI) can be selected (e.g., the nuclear compartment) and ε is calculated for each pixel. Finally, the average ε for that region is obtained by fitting to a Gaussian distribution. (C–D) BHK cells (transfected with pEGFP-GR or pEGFP) were incubated with vehicle (control), 100 nM dexamethasone (Dex), 100 nM Cort or 10 µM CpdA. Where indicated, ligand was removed by washing and changing to ligand-free media (washout) as previously described [27],[56]. For each cell (*n* = 273) ε was calculated as shown in (B). (C) Fold-increase of the nuclear brightness (ε) relative to the control (monomeric GR). Means ± SEM are shown. Bars with different superscript letters are significantly different from each other (*p*<0.05). (D) Subcelluar distribution in one representative cell for each treatment. Scale bar = 8 µm.

To determine the state of GR dimerization in living cells, we performed the N&B method [23]. This novel technique, based on moment-analysis, provides the average number of moving fluorescent molecules and their brightness at every pixel in the images (Figure 1A and 1B). In the simplest case the brightness of an oligomer consisting of *n* monomers is n-times the brightness of the n-monomers. Therefore, N&B is a useful method to obtain the oligomerization state of proteins in living cells with high spatial resolution. Figure 1C shows the nuclear brightness ε (i.e., measure of fluorophore oligomerization) corresponding to the wild-type enhanced green fluorescent protein (eGFP)-GR expressed in baby hamster kidney (BHK) cells. As we previously demonstrated [24], ε values significantly increased (approximately 2-fold) in the nucleus of cells treated with dexamethasone (Dex), consistent with a virtually complete population of GR dimers upon ligand addition. eGFP brightness is statistically indistinguishable from unstimulated eGFP-GR, indicating that nuclear eGFP-GR is mostly monomeric in the absence of ligand. Similar results were observed in the presence of the natural ligand corticosterone (Cort) (Figure 1C). As a negative control, no GR dimer formation was observed in cells treated with the non-steroidal ligand compound A (CpdA), in agreement with previous studies [25]. Recently, an *in vitro* study reported that the GR exists mostly as a monomer [26]. If that were the case *in vivo*, we would have detected an average brightness below two-fold in our system because of a linear-weighted-average combination from the contribution of the monomer/dimer population. Nevertheless, we cannot rule out the existence of a small population of monomeric molecules or even a small proportion of other oligomers.

Previous reports have suggested that GR dimer formation is an irreversible process *in vitro* [26]. Thus, we evaluated the stability of GR dimers *in vivo* by performing washout experiments. Interestingly, Cort withdrawal significantly reduced the population of GR dimers (Figure 1C), even though GR remained in the nucleus (Figure 1D), demonstrating that dimerization is a reversible process *in vivo*. Importantly, Dex washouts did not affect dimerization most likely due to the high affinity of this ligand for the receptor [27]. As we previously described [24], in our N&B assay there is an excess eGFP-GR molecules due to over-expression in comparison to accessible glucocorticoid response elements (GREs) at a given time. Moreover, any given GRE is only transiently bound by GR during physiological transcriptional activation [28],[29]. Hence, the virtually complete population of dimers observed is more compatible with a DNA-independent model for GR dimerization.

### The GRdim Forms Dimers In Vivo and Binds DNA

**Figure 2:**
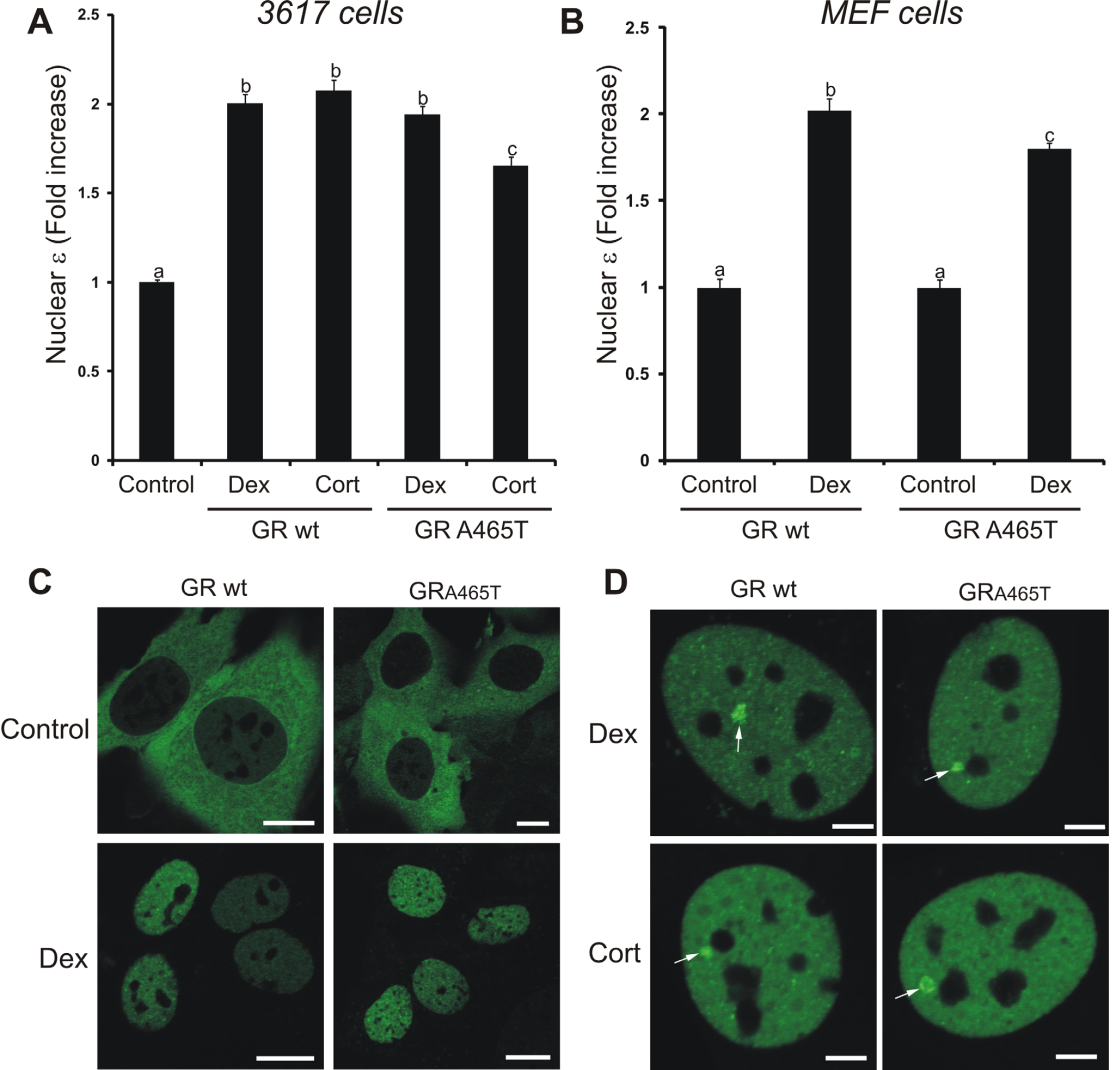
The GRdim forms dimers and binds DNA *in vivo*. 3617 cells transiently expressing eGFP-GRwt or eGFP-GR^A465T^ (A), or null GR MEF cell lines stably expressing eGFP-GRwt or eGFP-GR^A465T^ (B) were treated with vehicle (control), 100 nM Dex, or 100 nM Cort. The fold-increase of the nuclear brightness (ε) relative to the control (total *n* = 311) is shown. Bars with different superscript letters are significantly different from each other (*p*<0.05). (C) Subcelluar distribution of eGFP-GR in representative MEFs cells. Scale bar = 10 µm. (D) GR loading at the MMTV promoter array (white arrow) in the 3617 cell line (single cell analysis). Scale bar, 4 µm.

Next, we decided to test the oligomerization status of the GRdim mutant using N&B. Interestingly, treatment of eGFP-GR^A465T^ expressing cells with Dex showed an increase in nuclear brightness virtually identical to that observed with the wild-type receptor (Figure 2A), clearly demonstrating that this mutant is able to form dimers *in vivo*. Interestingly, the weaker natural steroid Cort also induces significant GR^A465T^ dimerization although with less efficiency than Dex (Figure 2A).

Recently, it has been suggested that GR expression levels affects the dimerization status of the receptor [30]. Since we were working in an over-expression system due to the transient transfection of eGFP-GR, we decided to study GR oligomerization status in a model expressing physiological levels of the receptor. We generated mouse embryonic fibroblast (MEF) cell lines from a GR null mouse stably expressing a mouse eGFP-GR protein at endogenous levels (Figure S1). N&B analysis of the MEF cell lines showed that the wild-type GR fully dimerizes in the presence of Dex and that the GRdim also forms dimers, although with a slightly less efficiency than the wild-type receptor (Figure 2B). Nuclear translocation was similar for both the GRwt and the GR^A465T^ mutant (Figure 2C). In conclusion, both ligand affinity and GR expression levels have no apparent effect on the dimerization status of the wild-type receptor. On the other hand, the GRdim oligomerization status is mildly sensitive to both ligand and receptor expression levels.

Another alleged property of the GRdim is its inability to bind DNA [16],[18], although it has also been questioned [19],[20],[22],[31]. To address this, we evaluated *in vivo* recruitment to DNA in the 3617 mouse cell line, which contains an amplified array of a GR responsive promoter structure (the mouse mammary tumor virus [MMTV] array). Thus, eGFP-GR interactions with MMTV GREs can be directly visualized in living cells as a bright spot [32]. Figure 2D clearly shows array formation on both GRwt and GR^A465T^ receptors. In summary, the GRdim seems to be able to dimerize and to bind DNA *in vivo*.

### Could the Dimeric GR also Be Responsible for Transrepression?

**Figure 3:**
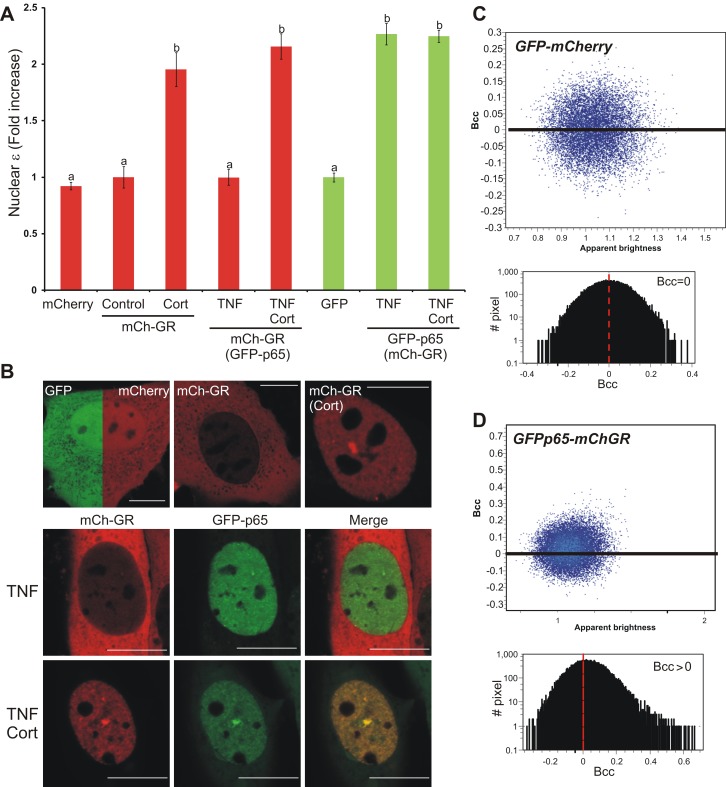
GR does not change its oligomerization state in the presence of NF-κB. 3617 cells transiently expressing the indicated combination of mCherry, mCherryGRwt, GFP, and/or GFP-P65 were treated with 300 nM Cort and/or 10 ng/ml TNF-α (TNF). (A) Fold-increase of the nuclear brightness (ε) relative to the control (total *n* = 215) for the green or red channels (color coded). Parenthesis means “in the presence of.” Bars with different superscript letters are significantly different from each other (*p*<0.05). (B) subcelluar distribution of eGFP of mCherry in one representative cell for each condition. Scale bar = 10 µm. (C–D) Cross correlation analysis of the fluorescence fluctuations. The cross correlation brightness (Bcc) plot for each pixel (blue dots) of a representative nucleus as defined in Digman and colleagues [35] as well as a histogram of the Bcc are shown.

The transcription factor NF-κB mediates key inflammatory pathways and its interaction with GR has been widely documented [1]. NF-κB is mainly composed of the heterodimer p50/p65, although p65 homodimers have also been described [33]. The transrepression hypothesis sustains that the GR interacts with p65 exclusively as a monomer; however, this idea relies almost entirely on the GRdim paradigm. The fact that monomeric GR molecules like CpdA-GR complexes are able to transrepress [34] does not rule out the possibility that GR dimers would also be capable of transrepression. To test this, we assessed the “dimeric transrepression” hypothesis by analyzing the oligomerization state of mCherry-GR in the presence of GFP-p65. Figure 3A shows N&B analysis of cells expressing mCherry or mCherryGR in the presence or absence of GPFp65, and Figure 3B contains representative images of these cells. As expected, in the presence of ligand mCherryGR showed full GR dimerization (Figure 3A). If GR interacts with p65 as a monomer, then GFP-p65 presence should decrease the population of mCherryGR dimers. However, no effect on mCherryGR oligomerization state is observed when GFP-p65 is present (Figure 3A). Brightness analysis also confirms that GFP-p65 dimerizes upon TNF addition. Moreover, this dimerization state was maintained in the nucleus containing Cort-activated GR molecules. To evaluate mCherryGR and GFP-p65 interactions, cross correlation analysis of the intensity fluctuations [35] was performed on the same data set. When untagged GFP and mCherry particles were analyzed, a symmetric cross correlation (brightness cross correlation [Bcc]) centered on zero was observed (Figure 3C), indicating an absence of interaction between the GFP-mCherry pair. On the contrary, mCherryGR and GFP-p65 showed an asymmetric, positive Bcc value (Figure 3C), indicating an interaction between GR and p65 molecules under our experimental conditions. Overall, although we cannot directly measure the stoichiometry of the GR-p65 complex, the most parsimonious model that fits our data is the one where Cort-stimulated GR is interacting with p65 as a dimer.

### Multiple Domains Are Involved in GR Dimerization

**Figure 4:**
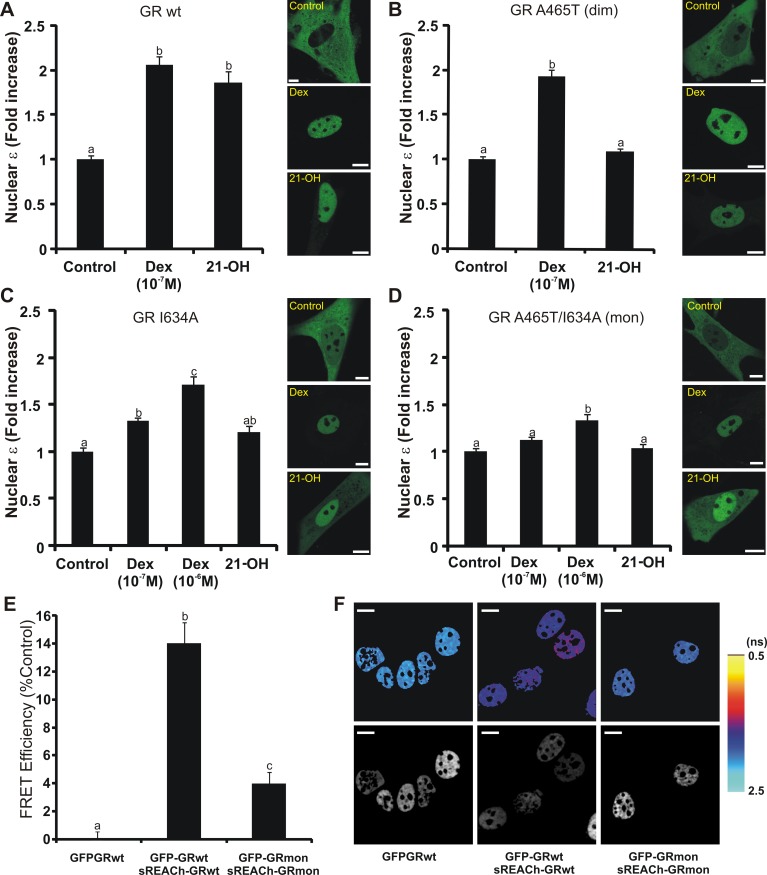
The GRmon is severely impaired in dimerization. (A–D) N&B assay. BHK cells (transfected with the indicated pEGFP-GR mutants) were treated with vehicle (control), Dex, or 10 µM 21-hydroxy-6,19-epoxyprogesterone (21-OH). The fold-increase of the nuclear brightness (ε) relative to the control (total *n* = 659) and the subcelluar distribution of eGFP-GR in one representative cell for each treatment are shown. Scale bar = 8 µm. (E–F) FLIM-FRET analysis. 3617 cells transiently transfected with the indicated plasmids were treated with 100 nM Cort for 30 min and fixed. The lifetime of EGFP was measured as described in the “Materials and Methods” section. The mean FRET efficiency for the *sREACh-EGFP* pairs ± SEM (*n* = 25–26 cells per group) (E) and representative cells (F) are shown; with pseudo-color-coded lifetime images scaled from 0.5–2.5 ns where warmer colors represent shorter liftetime values (top row) and the corresponding fluorescence intensity images (bottom row). Scale bar = 10 µm. Distribution frequencies of lifetime maps are additionally presented in Figure S3.

As demonstrated above, GRdim is able to form dimers *in vivo* (Figure 2). If the DBD dimerization surface is indeed compromised in the GRdim mutant, then another region of the protein must participate in GR-GR interactions. An interesting candidate is the LBD region, where a second dimerization surface has been described [15] but whose functional relevance has been questioned on the basis of studies performed with DBD mutants like GRdim [10],[36]. According to the GR LBD/Dex crystal structure, the dimerization interface includes a central hydrophobic region made up of reciprocal interactions between residues in the βA strand and a network of hydrogen bonds involving residues of the H1–H3 loop [37]. In a previous report, we characterized a rigid steroid ligand, 21-hydroxy-6,19-epoxyprogesterone (21OH-6,19OP), which behaves as a GR agonist in transrepression assays but as an antagonist in transactivation ones [24]. According to molecular dynamics (MD) simulations, 21OH-6,19OP induces a dramatic change in the average position of the H1–H3 loop within GR's LBD [38]. N&B studies showed that 21OH-6,19OP is still able to induce GR dimerization (Figure 4A and [24]), which suggests that GR-21OH-6,19OP complexes dimerize through the DBD dimerization surface since the H1–H3 loop is compromised. Consistent with this hypothesis, GR^A465T^ dimerization was abrogated in cells treated with 21OH-6,19OP (Figure 4B), even though this compound induced GR nuclear translocation (Figure 4B, right panel). Together, these results suggest that GR form dimers *in vivo* through the combined action of the LBD and the DBD regions. This model explains how the MD predictions performed on the GR-21OH-6,19OP complex are only detected *in vivo* when the DBD is compromised (i.e., in cells expressing GR^A465T^).

### The GRmon: A Monomeric Glucocorticoid Receptor

To further evaluate the functional contribution of the DBD and LBD regions on GR dimerization, we constructed the mutant eGFP-GR^I634A^, on the basis of the orthologous human mutation I628A (residue localized at the βA strand) previously reported to decrease by 10-fold the dimerization of LBD-LBD fragments *in vitro* [15]. Figure 4C shows that eGFP-GR^I634A^ has a diminished ability to form dimers in the presence of 0.1 µM Dex, although its subcelluar localization remains nuclear (Figure 4C, right panel). Activation of the receptor with 1 µM Dex slightly increases dimerization, supporting a previous report suggesting that the human I628A may have reduced affinity for Dex [15]. Interestingly, when we combined the mutations in the DBD and the LBD dimerization surface (eGFP-GR^A465T/I634A^) dimer formation was completely abolished with 0.1 µM Dex and severely compromised in the presence of 1 µM Dex (Figure 4D), suggesting a combinatorial contribution of both domains on GR dimerization. Similar behavior of these mutants was observed in both 3617 cells and in the MEF cell line with low-expression levels of GR (Figure S2). Accordingly, we named the GR^A465T/I634A^ mutant GRmon as it is defective in dimerization *in vivo*. Consistent with the N&B data, the Förster resonance energy transfer (FRET) assay also indicated that the GRmon is impaired in dimerization (Figures 4E, 4F, and S3).

**Figure 5:**
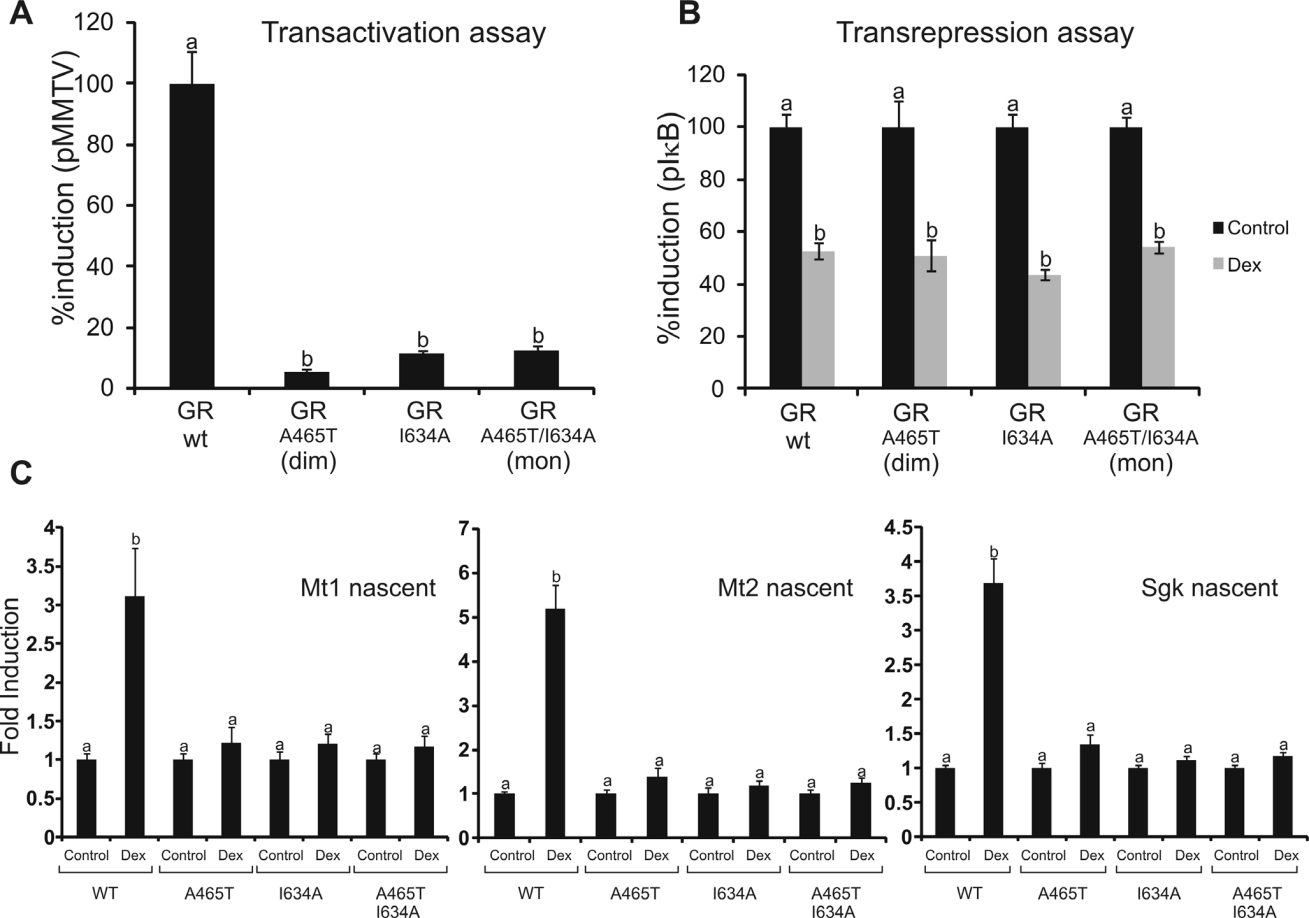
Transcriptional activity of the GR mutants. (A–B) Cos-7 cells were co-transfected with pEGFP-GR vectors and the MMTV-Luciferase reporter vector (A) or pkB-Luciferase reporter and pRelA expression vectors (B). Cells were incubated in the presence or absence of 100 nM Dex and luciferase activity was measured. (A) Values (induction efficiency) were expressed as percentage activity relative to Dex-treated wild-type GR. (B) Values (GR's inhibition efficiency on NF-κB activity) were expressed as percent induction relative to the control. (C) Nascent mRNA levels on MEFs cell lines stably expressing the GFPGR mutants. Means ± SEM from three independent experiments are shown. Bars with different superscript letters are significantly different from each other (*p*<0.05).

We next characterized the transcriptional activities of all GR mutants. In agreement with previous data [16] luciferase reporter assays showed that GR^A465T^ has little transactivation activity (Figure 5A) but similar transrepression efficiency (Figure 5B) compared to the wild-type receptor. As originally reported [15], GR^I634A^ also promotes poor transactivation activity (Figure 5A); however, transrepression of an NF-κB reporter was not affected (Figure 5B). The GRmon behaves similarly to the single DBD and LBD point mutants (Figure 5). Consistently, transcriptional activation of endogenous genes in the eGFPGR-MEFs cell lines showed a similar trend (Figure 5C).Taken together, our results show no correlation between the dimeric/monomeric state of the receptor and its ability to transactivate or transrepress gene expression, at least in the context of reporter gene assays.

### GR Recruitment to GREs: De Novo Versus Pre-programmed Sites

**Figure 6:**
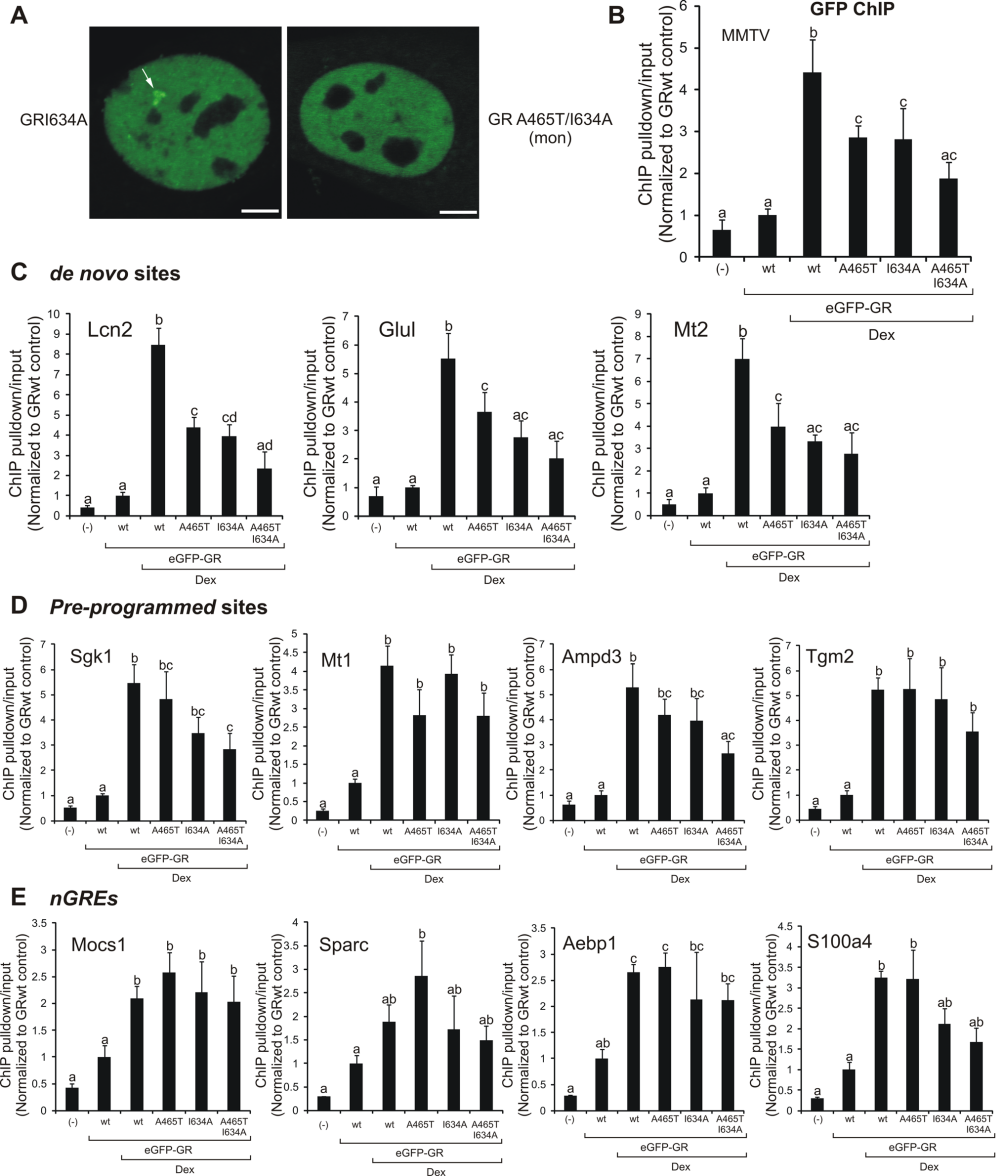
DNA binding and chromatin recruitment of the GR mutants. (A) GR loading at the MMTV promoter array (white arrow) in the 3617 cell line (single cell analysis). Scale bar, 4 µm. (B–E) ChIPs using a GFP antibody in the 3134 cell line previously transfected with pEGFP-GR mutants. qPCR data as ChIP pulldown/input normalized to vehicle-treated cells (*n* = 4) for the MMTV promoter array (B) or endogenous GR binding sites, either *de novo* (C), pre-programmed (D), or nGRE (E). Means ± SEM from four independent experiments are shown. Bars with different superscript letters are significantly different from each other (*p*<0.05). If at least one superscript letter is shared between treatments, then no significant differences were found.

We next analyzed the ability of the LBD mutants to bind to the MMTV array in 3617 cells. Similar to the GRwt and GR^A465T^ (Figure 2D), array formation was successfully observed upon Dex addition with GR^I634A^ (Figure 6A, white arrows). On the contrary, although a few cells were positively visualized (unpublished data), we failed to observe a considerable number of cells with arrays in the presence of GRmon (Figure 6A). To confirm these results with an average-population, quantitative approach, we performed chromatin immunoprecipitation (ChIP) assays using a GFP antibody on the MMTV array. In agreement with the imaging data, all single GR mutants can occupy the MMTV region but GRmon is poorly recruited (Figure 6B). Interestingly, both GR^A465T^ and GR^I634A^ are able to bind DNA with less efficiency than their wild-type counterpart (Figure 6B). The eGFP-GR mutants in ChIP experiments were expressed at a similar level (Figure S4).

The recruitment of transcription factors to chromatin depends on a variety of complex events. An emerging paradigm suggests that the local chromatin structure of response elements contributes strongly to the tissue-specific action of many transcription factors [39]. In particular, *in vivo* GR recruitment to DNA is strongly dependent on the chromatin landscape, with most of the GR binding events occurring at pre-programmed chromatin (i.e., DNaseI hypersensitive sites prior to ligand treatment) and only a small fraction of binding at *de novo* sites (i.e., DNaseI sites actively induced by the receptor) [40]. To further characterize the GR mutants, we performed ChIP assays on a few pre-programmed or *de novo* sites. Irrespective of the sites analyzed, both the GR^A465T^ and the GR^I634A^ were able to bind chromatin (Figure 6C and 6D), although their relative occupancy compared to the wild-type was site-specific. Interestingly, GRmon was recruited to most of the pre-programmed sites evaluated (Figure 6D) while no significant binding was observed to *de novo* sites (Figure 6C). Finally, we evaluated GR recruitment to recently reported negative GREs (nGREs) [41], which were suggested to be preferential binding sites of the monomeric GR [9]. To identify nGREs in 3134 cells, we overlapped all 1,147 putative nGREs conserved between human and mouse [41] with GR ChIP-seq data from 3134 cells [40]. Surprisingly, only five were found at GR binding sites in 3134 cells (Figure S5). From these, three were located near Dex-repressed genes as shown by microarray analysis [42]. ChIP results show no clear link between the mutants and the receptor's ability to bind nGREs (Figure 6E). In summary, our data suggest that the dimeric status of the receptor neither defines its transactivation activity nor predicts its ability to bind chromatin *in vivo*. On the other hand, the monomeric form of GR seems to be less efficient in its ability to bind chromatin than the dimeric form of the receptor.

## Discussion

Studies mainly using the GRdim mutant suggested the dissociated model of GR action and led to the transrepression hypothesis [2]. This hypothesis states that suppression of inflammation by GR is mainly mediated by the transrepression mechanism, and is independent of GR transcriptional regulation through its direct binding to DNA. Accordingly, side effects of glucocorticoids were suggested to be dependent on GR dimerization, GR-GRE interaction, and the downstream consequence on gene regulation. This model has been the guiding principle in the search of new compounds with dissociated glucocorticoid properties [11]. Today this strategy is deeply criticized, not only because it is known that some glucocorticoid anti-inflammatory effects depend on gene activation [2],[43], but also because evidence against GRdim's alleged monomeric status and inability to bind DNA is accumulating [19],[20],[22],[31]. Here, we demonstrate that the so-called GRdim is able to dimerize *in vivo* while the new mutant GRmon (A465T/I634A) is severely impaired in dimer formation. We have studied the oligomerization state of GR by the novel N&B technique, under both physiological and over-expressed GR levels. Independent confirmation that the GRmon is impaired in dimerization has been obtained by fluorescence lifetime imaging microscopy (FLIM)-FRET analysis.

If the GRdim is still able to bind DNA and form dimers as demonstrated here and elsewhere [19],[20],[22],[31], why is this mutant unable to transactivate genes? Recent studies have shown that the GRdim's residence time on DNA is ten times less than the one observed for wild-type GR [29], in strict agreement with its diminished transcriptional activity according to the “hit and run” model of transcriptional activation [44],[45]. Also, it has been shown *in vitro* that the dim mutation alters the allosteric effect that DNA exerts on GR, therefore varying the receptor's conformational states and perhaps changing the ability to interact with co-regulators [20]. Even though it has not been directly tested, GRdim's altered ability to interact with specific cofactors could explain why this mutant is able to induce the expression of genes whose promoters contain certain GREs and not others [19]–[21]. In other words, the dim mutation does not actually appear to abolish GR transactivation altogether but instead their effect depends on both gene and cellular context, producing an overall change in the whole transcriptional outcome. As an example, a microarray analysis performed in U-2 OS cells showed a very different pattern of gene regulation comparing wild-type and GRdim expressing cells [22]. Moreover, expression analysis performed in livers from wild-type and dim mice revealed that GRdim could induce gene expression when compared with wild-type GR [46]. Overall, there is compelling evidence that suggests that the transactivation versus transrepression model that arouse from the GRdim mouse phenotype was oversimplified and needs re-examination [2],[43]. More genome-wide studies on the dim model will provide much needed insights in the mechanisms underlying the GRdim mice phenotype.

The establishment in the community that transactivation is mediated by GR dimers and transrepression occurs exclusively through GR monomers has been built almost entirely under the GRdim paradigm [16],[18],[47]. However, here we find no correlation between the dimeric/monomeric state of the receptor and its ability to transactivate or transrepress reporter genes. For example, even though GRwt and GRdim are mainly dimeric the latter is severely impaired in transactivation compared to the wild-type GR. On the other hand, the GRmon is mainly monomeric but its transrepression efficiency is indistinguishable from the fully dimeric wild-type receptor. Hence, changing the relative population between dimers and monomers does not necessarily change the transcriptional outcome. In conclusion, GR dimerization appears necessary but not sufficient for transactivation and it is not required for transrepression. Nonetheless, given the fact that GR transcriptional activity is highly gene- and cell type- specific more studies are needed to properly evaluate the scope of this conclusion. Interestingly, our data suggest that Cort-GR molecules remain dimeric in the presence of GR/NF-κB interactions. Thus, the idea that transactivation could be dissociated from transrepression through manipulation of the oligomerization state of the receptor should be critically revised, if not entirely discarded.

Overall, our results indicate that GR dimerization involves a more complex mechanism than previously anticipated. Moreover, we also challenge the view that transrepression is exclusively performed by the monomeric GR. This implies that the simplified monomer/dimer model equilibrium does not explain GR transactivation versus transrepression activity. It seems that the prevailing view was established without rigorous verification and new approaches for mitigating the side effects of chronic glucocorticoid treatment should be explored.

## Materials and Methods

### GR Ligands

Dex and Cort were purchased from Sigma-Aldrich. CpdA [25] was purchased from Enzo Life Sciences. 21OH-6,19OP was prepared as previously described [48].

### Plasmids Constructs

pEGFP-GR expresses the eGFP protein fused to the N-terminal end of the mouse GR [24]. pEGFP-GR^A465T^ was generated by site-directed mutagenesis by TOP Gene Technologies. pEGFP-GR^I634A^ and pEGFP-GR^A465T/I634A^ were generated by site-directed mutagenesis by Stony Brook cloning facility (Stony Brook University, New York, USA). mCherry-GR was previously described [49]. For FLIM-FRET experiments, the coding region of the super (s)REACh fluorophore [50] was subcloned into the N-terminal of the mouse GR sequence (psREACh-GR). Briefly, the AgeI-BglII eGFP containing sequence of pEGFP-GR and pEGFP-GR^A465T/I634A^ was replaced with sREACh cDNA PCR amplified from mGFP-10-sREACh-N3 (Addgene, plasmid 21947) using the Herculase II fusion DNA polymerase system (Agilent Technologies). The reverse primer contained an additional five bases, introducing 5′-TACTC-3′ into the plasmid prior to the BglII restriction site and so preserving the same linker as the eGFP variants.

pMMTV-luciferase; pkB-luciferase, pRelA and pCMV-LacZ were previously described [24]. GFP-p65 was a kind gift from Alessandra Agresti [51]. The SV40T-expressing retroviral pBabe-largeT cDNA and the retroviral pWZL-neo plasmids were a gift from Kai Ge [52]. The coding region of the eGFP-GR mutants was cloned into the pWZL-neo vectors for retroviral transduction. Briefly, each eGFP-GR coding sequence was independently isolated by PCR (using the high fidelity Herculase II polymerase) with primers carrying BamHI and MfeI restriction sites: forward (For) atatggatccGTGAACCGTCAGATCCGCTAG and reverse (Rev) atcgCAATTGGGCAGCCTTTCTTAGTAAGGCAG. The purified fragment was subcloned into BamHI/MfeI sites of the pWZL-neo vector.

### Cell Culture

BHK21 and Cos-7 cells were cultured in DMEM (Invitrogen) supplemented with 10% FBS (Internegocios S.A.). 3134 and 3617 cells were cultured in DMEM and supplemented with 10% FBS (Hyclone). The 3134 cell line is a mouse mammary adenocarcinoma cell line. It contains a large tandem array (∼200 copies) of a mouse mammary tumor virus, Harvey viral ras (MMTV-v-Ha-ras) reporter. The 3617 cell line is a derivative of 3134 cell line expressing a GFP-tagged version of GR (GFP-GR) from a chromosomal locus under control of the tetracycline repressible promoter. Both cell lines were described previously [53]. In all cases, prior to glucocorticoid treatment cells were incubated at least 18 h in DMEM medium containing 10% charcoal-stripped FBS (Hyclone).

### GFP-GR Mutants-MEFs Cell Line

Heterozygous GR-deficient (GR het) mice were generated by crossing mice with one allele of GR exon 3 flanked by loxp sites [54] with mice expressing Cre driven by the b-actin promoter. Day 13.5 embryo bodies from a timed GR het×GR het mating were minced with scissors and forceps, digested with trypsin, and cultured in DMEM supplemented with FCS and glutamine at 37°C in 5% CO2. GR-deficient MEFs were identified by PCR as being positive for the deleted allele and negative for the germline allele. Primary fibroblasts were immortalized via retroviral transduction with SV40 large T antigen. Briefly, 5 million Phoenix A cells were plated in a 10-cm dish 24 hours prior to transfection with 10 µg pBabe-SV40 (Puro) plasmid using JetPRIME transfection reagent (Polyplus transfection) according to the manufacturer's recommended protocol. Virus containing supernatant was collected 48 hours post-transfection and filtered through a 0.45 µM filter. Filtered virus-containing Phoenix cell supernatant was diluted with an equal volume of fresh media and polybrene was added to a final concentration of 5 µg/ml. 2 ml of this virus solution was used to infect 200,000 MEFs. 48 hours post-transduction the cells were challenged with 2 µg/ml puromycin (SIGMA-Aldrich). Puromycin selection was complete in 3–4 days, however these large T antigen immortalized MEFs were maintained in media containing 2 µg/ml puro. The immortalized MEF cell lines (wt and GR−/−) were transduced with pWZL-GFPGR (Neo) as described above. These cells were selected with 500 µg/ml G418 (Cellgro). After 15 days of Neomycin selection, cells were sorted by FACS according to their GFP expression into three categories (low, medium, high). eGFP-GR levels were monitored by Western blot (Figure S1) and medium expression cells were chosen for further studies.

### Transient Transfections

BHK21 and Cos-7 cells were transiently transfected with Lipofectin 2000 (Invitrogen) according to manufacturer's instructions. 3134 and 3617 cells were transfected with jetPRIME reagent (VWR) according to manufacturer's instructions.

### Subcellular Localization and N&B Analysis

3×10^5^ BHK cells were transfected with 1.5 µg of pEGFP-GR or the mutant variants and incubated with vehicle, 100 nM Dex, 1 µM Dex, 100 nM Cort, 10 µM 21OH-6,19OP, or 10 µM CpdA for at least 1 h. Washout procedures consisted in washing the cells three times with pre-warmed (37°C) PBS and then adding hormone-free media for 20–40 minutes before analysis. Measurements were done in a FV1000 confocal laser scanning microscope (Olympus), with an Olympus UPlanSApo 60× oil immersion objective (NA = 1.35). The excitation source was a multi-line Ar laser tuned at 488 nm (average power at the sample, 700 nW). Fluorescence was detected with a photomultiplier set in the pseudo photon-counting detection mode.

3617 cells were grown in the presence of 5 µg/ml tetracycline (Sigma-Aldrich) to inhibit the stable GFP-GR gene expression [27],[53], and transiently transfected with 1.5 µg of pEGFP-GR or the mutant variants, or a combination of mCherryGR and GFP-p65 as indicated. Cells were incubated for at least 30 min with 100 nM Dex, 100 nM Cort, or 300 nM Cort in the presence or absence of 10 ng/ml TNFα (Sigma-Aldrich). Measurements were done in a LSM 780 laser scanning microscope (Carl Zeiss, Inc.) at the CCR Confocal Microscopy Core Facility (NIH, Bethesda, Maryland, USA). We used a 63× oil immersion objective (NA = 1.4). The excitation source was a multi-line Ar laser tuned at 488 nm and or a 594 nm laser. Fluorescence was detected with a GaAsP detector in photon-counting mode.

N&B measurements were done as previously described [23] with some modifications [24]. Briefly, for each studied cell a stack of 150–200 images (256×256 pixels) were taken in the conditions mentioned above, setting the pixel size to 80–82 nm and the pixel dwell time to 6.3 or 10 µs. Each stack was further analyzed using the N&B routine of the “GLOBALS for Images” program developed at the Laboratory for Fluorescence Dynamics (UCI, Irvine, California, USA). In this routine, the average fluorescence intensity (<I>) and its variance (σ^2^) at each pixel of an image are determined from the intensity values obtained at the given pixel along the images stack. The apparent brightness (B) is then calculated as the ratio of σ^2^ to <I> while the apparent number of moving particles (N) corresponds to the ratio of <I> to B. In a previous work it has been demonstrated that B is equal to the real brightness ε of the particles plus one [23]. Therefore, ε at every pixel of images can be easily extracted from B measurements. Importantly, this analysis only provides information regarding the moving or fluctuating fluorescent molecules since fixed molecules will give B values equal to 1.

### Transactivation and Transrepression Assays

For the transactivation assay, 3×10^5^ Cos-7 cells were co-transfected with 1.5 µg pMMTV-luciferase vector and 0.5 µg of pEGFP-GR vectors. For the NF-κB transrepression assay, 1.5 µg pkB-luciferase and 1.5 µg pRelA were used. In all cases, 0.5 µg pCMV-LacZ was added as transfection control. After transfection, cells were incubated in DMEM containing 5% charcoal-stripped FBS and incubated with 100 nM Dex for at least 18 h. Luciferase activity and β-galactosidase activity was measured as previously described [24].

### Fluorescence Lifetime Imaging Microscopy: Förster Resonance Energy Transfer

3617 cells were seeded to 22×22 mm glass coverslips in six-well tissue culture plates. Media contained 10% charcoal-stripped serum and tetracycline (5 µg/ml) to prevent GFP-GR expression. Next day cells were transiently transfected with 2 µg total plasmid using JetPRIME (VWR) and the manufacturer's protocol. EGFP (donor) and sREACh (acceptor) plasmids were transfected at 1∶2 ratio to maximize the chances of seeing an interaction by FRET. 24 h after transfection cells were treated for 30 min with 100 nM Cort and fixed in paraformaldehyde added to media (4% final concentration) for 15 min. Coverslips were washed 3× in PBS and mounted to microscope slides with Mowiol 4–88 containing 1 mg/ml p-phenylenediamine as anti-fade (both Sigma-Aldrich). Images were acquired on a Leica DMI 6000 SP5 inverted confocal microscope with a 63× oil immersion objective of NA 1.4 (Leica Microsystems). EGFP excitation at 850 nm was achieved with a femtosecond mode-locked (80 MHz repetition rate) Mai-Tai HP pulsed, multi photon laser (Spectra Physics). Fluorescence was collected using a HPM100 Hybrid Detector R3809U-50 (Becker & Hickl; Hamamatsu Photonics) through a band-pass GFP filter at ET 525/50 (Chroma Technology Corp). Fluorescence decays were resolved by time-correlated single-photon counting (TCSPC) using a SPC830 acquisition board (Becker & Hickl). Images were acquired in 256×256 pixel format collecting at least 1,000 photons per pixel over 2–5 min. Fluorescence transients were acquired with SPCImage software (Becker & Hickl), analyzed according to single-life time decay, then exported to Image J (NIH). An in-house Image J protocol permitted selection of the relevant pixels (nucleus) and derivation of histograms for the weighted mean average of the fluorescent lifetimes. These were plotted as frequency distributions normalized and integrated for area under the curve using Igor Pro (WaveMetrics Inc). The weighted mean lifetime (T) was extracted from histograms of individual cells in Image J and converted to FRET efficiency relative to the GFP-GR control according to: FRET Efficiency (%) = 1−(Tdonor/Tdonor+acceptor)−100 to allow statistical analysis.

### Chromatin Immunoprecipitation

3134 cells were seeded in 150 mm tissue culture plates and the next day transiently transfected with 10 µg of pEGFP-GR or the mutant variants. Cells were collected the next day after 1 h of 100 nM dex treatment. ChIP was performed according to the standard protocol (Upstate Biotechnology) with a crosslinking step (1% formaldehyde at RT), followed by a quenching step with 125 mM glycine. Chromatin was sonicated by using the Bioruptor sonicator (Diagenode) with 15 s “on” and 15 s “off” for 30 cycles. Sonication efficiency was monitored by 2% agarose gel electrophoresis. Sonicated chromatin (400 µg) was immunoprecipitated with an antibody against GFP (Abcam ab290). DNA isolated from immunoprecipitates, as well as input DNA, was used as a template for real-time PCR (qPCR). Primers used for qPCR are (5′→3′): MMTV, For TGGTTACAAACTGTTCTTAAAACGAGGATG and Rev CTCAGATCAGAACCTTTGATACCAAACC; LCN2, For TCACCCTGTGCCAGGACCAA and Rev TGGGGAAGGGTGAGCAAGCT; GluL, For CACTTGGGCAAACATGGACGGT and Rev CACAAGAGGAAATGCCCCCCT; Mt2, For CATAGCCAGGGCAGCCACAGAA and Rev GGCAATGCCTTCTTGACTCATTCC; SGK, For CACTTGGGCAAACATGGACGGT and Rev CACAAGAGGAAATGCCCCCCT; Mt1, For TAGGGACATGATGTTCCACACGTC and Rev TTTTCGGGCGGAGTGCAGAG; Tgm2, For CCACACATTGGTTTTGCTATGCTTG and Rev AATCATTTTCTCATTCCACACAGCC; Ampd3, For GCCAGGACGTGGTGTTCAGGAT and Rev GGGCTGGAAATTCTCCTGCG; Sarc, For CCTCAGTCAGTGCTCAGTGG and Rev GGGACCAGATGGGATATCAG; Aebp1, For CTCTTATGCAATCGTTGTCAGTAAATCT and Rev ATGATGAATGGTGCCTTACAGTCTC; Mocs1, For ATTTGGCAGAGACTAGCCTGGAAATGAT and Rev CATCTTATGACCTACTTCCACCCCA; S100a4, For ATGGGGTAAGGAGCGGAAGG and Rev CTGGACCCAGCCATGCCCTC. Standard curves were created by 4-fold serial dilution of an input template. The data presented are from four independent experiments.

### Reverse Transcriptase-qPCR

The MEFs cell lines were plated for 48 h in DMEM medium containing 10% charcoal-stripped FBS and then treated for 1 h with 100 nM Dex. RNA extraction was performed with the Nucleospin RNA-kit (Clontech) according to manufacturer's instructions. cDNA was made with the iScript cDNA Synthesis Kit (Bio-Rad Laboratories, Inc.) from 1 µg RNA. Upon dilution, cDNA was subjected to qPCR using the iQ SYBR green supermix (Bio-Rad) with the indicated primers. Primer sequences were designed to amplify only nascent RNA, using PCR amplicons that cross an exon/intron or UTR/intron boundary. Primer sequences are as follows: Mt1, For CCTCACTTACTCCGTAGCTCCAGC and Rev TCCCGCCAAGCCTCTACAACTC; Mt2, For GAACTCTTCAAACCGATCTCTCGTC and Rev TCCCAGAAATCCCGTCAGCA; SGK, For GGGAATGGTAGCGATTCTCATCG and Rev CGACGCCACACGCTAATCTG; Actin, For AGTGTGACGTTGACATCCGTA and Rev GCCAGAGCAGTAATCTCCTTCT.

### Western Blot Analyses

Chromatin samples from ChIPs experiments (i.e., inputs) were separated by SDS–PAGE and transferred to PVDF membranes. Blots were probed with primary antibodies anti-GR (sc-1004; 1∶1,000), anti-actin (sc-1615; 1∶1,000) (Santa Cruz Biotechnology), or anti-GAPDH (Abcam, ab-8245, 1∶1,000) in Tris-buffered saline (TBS) containing 5% nonfat dry milk, followed by incubation with horseradish peroxidase (HRP)-conjugated anti-goat, anti-mouse, or anti-rabbit antibody (Santa Cruz Biotechnology). All blots were visualized with the ECL kit (Supersignal).

### Statistical Analysis

Results were expressed as means ± SEM. Statistical analyses were performed with STATISTICA 7.0 (StatSoft, Inc.) and consisted of one-way ANOVA followed by Tukey's multiple comparisons tests. Differences were regarded as significant at *p*<0.05 (bars with different superscript letters are significantly different from each other). Before statistical analysis, data were tested for homoscedasticity using Bartlett's test. In some cases, transformation of the variable (x′ = √x) were necessary.

## Supporting Information

Figure S1 **Generation and characterization of the MEFs cell lines.** MEF cells were obtained from GR null mice (A) and immortalized by transduction with a retrovirus expressing the SV40 large T antigen (B). Next, the established MEF GR knock-out cell line was transduced with the eGFP-GR mutants and selected for Neomycin resistance (C). Finally, each cell line was sorted by FACS according to their GFP levels (D). The “medium” expression showed similar eGFP-GR levels to the endogenous GR in wild-type MEFs (E). Thus, these cell lines were chosen for all further experiments. Western blot analysis during the entire MEF generation procedure is shown. For more detail please see the “Materials and Methods” section.
(TIF)

Figure S2 **Number and brightness assay on 3617 and MEF cells.** 3617 cells transiently expressing the eGFP-GR mutants (A), or null GR MEF cell lines stably expressing the eGFP-GR mutants (B) were treated with vehicle (control), 100 nM Dex, or 100 nM Cort. The fold-increase of the nuclear brightness (ε) relative to the control (total *n* = 277 for 3617 cells and *n* = 164 for the MEF cells) is shown. Bars with different superscript letters are significantly different from each other (*p*<0.05). (C) Subcelluar distribution of eGFP-GR in representative MEF cells (C). Scale bar = 10 µm.
(TIF)

Figure S3 **FLIM-FRET analysis on GRwt and GRmon.** 3617 cells were transiently transfected with the indicated combination of plasmids and treated for 30 min with 100 nM Cort. Frequency distribution for the eGFP photon lifetimes with different donor-acceptor combinations (*n* = 25–26 cells per condition) is shown. The constructs are identical aside from the indicated mutations in GR and the fluorophore tag. eGFP-GRwt/sREACh-GRwt shifts to reduced lifetimes values relative to the control (no FRET) indicating interaction of the alternatively tagged GRwt proteins. In contrast, the higher lifetime values for eGFP-GRmon/sREACh-GRmon indicate impaired interaction examined by FRET. The binomial distribution of lifetimes in the eGFP-GRwt/sREACh-GRwt pairing could be due to different FRET efficiencies produced by varied transient transfection of donor to acceptor across the cell population.
(TIF)

Figure S4 **eGFP-GR protein levels in ChIPs experiments.** Chromatin extracts (inputs) from ChIP experiments in Figure 3 were subjected to Western blot analysis in order to monitor expression levels between the different eGFP-GR variants. (A) Representative Western blot using an anti-GR or anti-actin antibody. (B) Normalized eGFP-GR levels (mean ± SD) from four independent experiments.
(TIF)

Figure S5 **Searching for nGRE in 3134 cells.** (A) Venn diagram generated by overlapping all putative nGREs conserved between human and mouse (taken from Surjit et al., Table S1 [34]) with GR ChIP-seq data (taken from John et al. [33]). (B) The table shows the genome localization (mm9) of each nGRE found (named after the nearby gene). We also show the relative expression according to previously published microarray data [35]. (C) Genome browser shots of the nGREs from GR ChIP-seq data [33].
(TIF)

## Abbreviations

21OH-6,19OP, 21-hydroxy-6,19-epoxyprogesterone</expn>
Bcc: brightness cross correlation
BHK: baby hamster kidney cells
ChIP-seq: chromatin immunoprecipitation followed by deep-sequencing
Cort: corticosterone
CpdA: compound A
DBD: DNA-binding domain
Dex: dexamethasone
FLIM: fluorescence lifetime imaging microscopy
For: forward
FRET: Förster resonance energy transfer
GR: glucocorticoid receptor
GRE: glucocorticoid response element
LBD: ligand-binding domain
MEF: mouse embryonic fibroblast
MD: molecular dynamics
MMTV: mouse mammary tumor virus
N&B: number and brightness
nGRE: negative GRE
Rev: reverse
