# Supplementary figures and images for: Live Cell Imaging Unveils Multiple Domain Requirements for In Vivo Dimerization of the Glucocorticoid Receptor
Source: PLoS Biol. 2014 Mar 18;12(3):e1001813. doi: 10.1371/journal.pbio.1001813 (PMC3958349; doi:10.1371/journal.pbio.1001813)

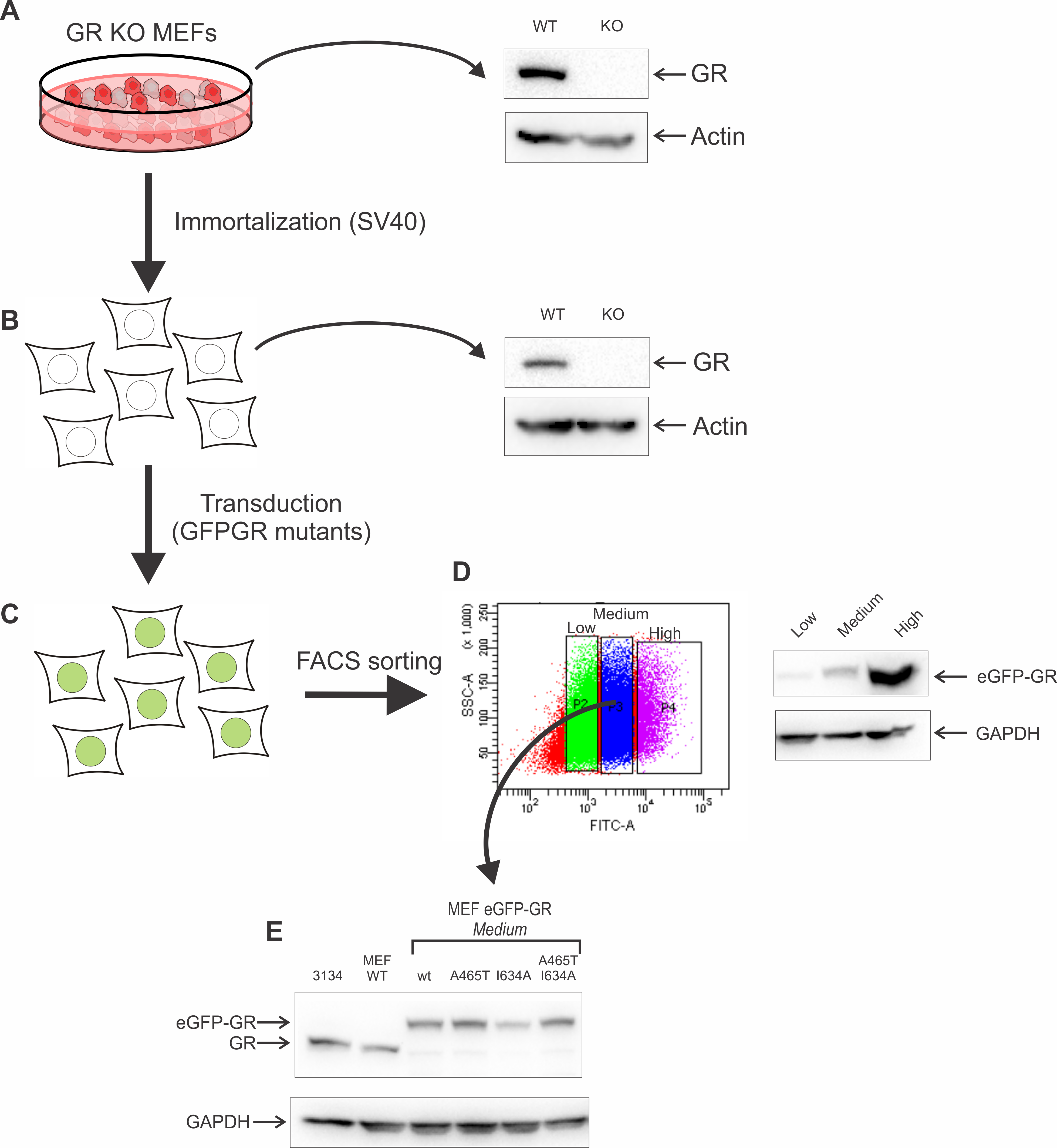

Supplement: Figure S1 — Generation and characterization of the MEFs cell lines. MEF cells were obtained from GR null mice (A) and immortalized by transduction with a retrovirus expressing the SV40 large T antigen (B). Next, the established MEF GR knock-out cell line was transduced with the eGFP-GR mutants and selected for Neomycin resistance (C). Finally, each cell line was sorted by FACS according to their GFP levels (D). The “medium” expression showed similar eGFP-GR levels to the endogenous GR in wild-type MEFs (E). Thus, these cell lines were chosen for all further experiments. Western blot analysis during the entire MEF generation procedure is shown. For more detail please see the “Materials and Methods” section. (TIF) [file pbio.1001813.s001.tif]

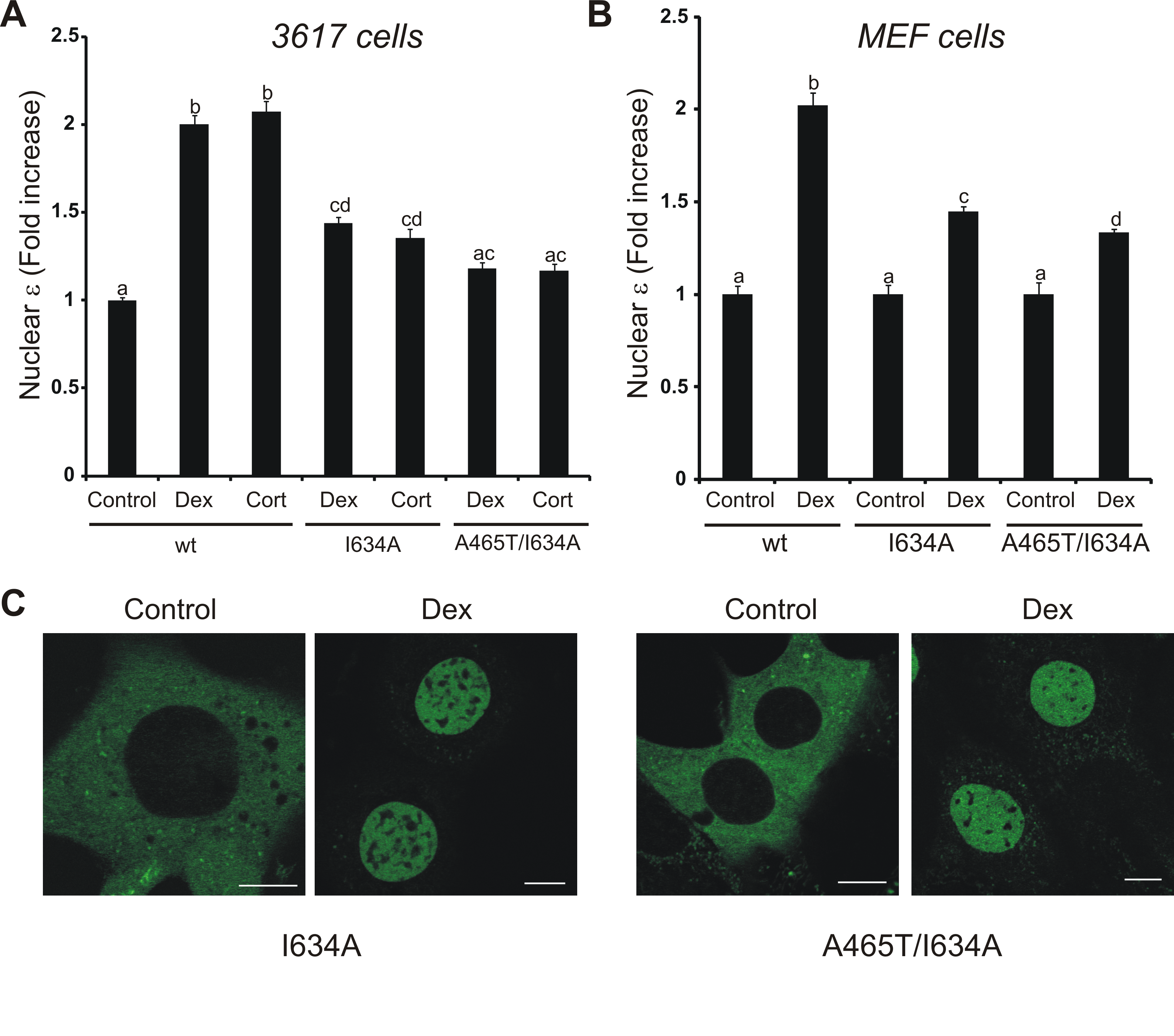

Supplement: Figure S2 — Number and brightness assay on 3617 and MEF cells. 3617 cells transiently expressing the eGFP-GR mutants (A), or null GR MEF cell lines stably expressing the eGFP-GR mutants (B) were treated with vehicle (control), 100 nM Dex, or 100 nM Cort. The fold-increase of the nuclear brightness (ε) relative to the control (total n = 277 for 3617 cells and n = 164 for the MEF cells) is shown. Bars with different superscript letters are significantly different from each other (p<0.05). (C) Subcelluar distribution of eGFP-GR in representative MEF cells (C). Scale bar = 10 µm. (TIF) [file pbio.1001813.s002.tif]

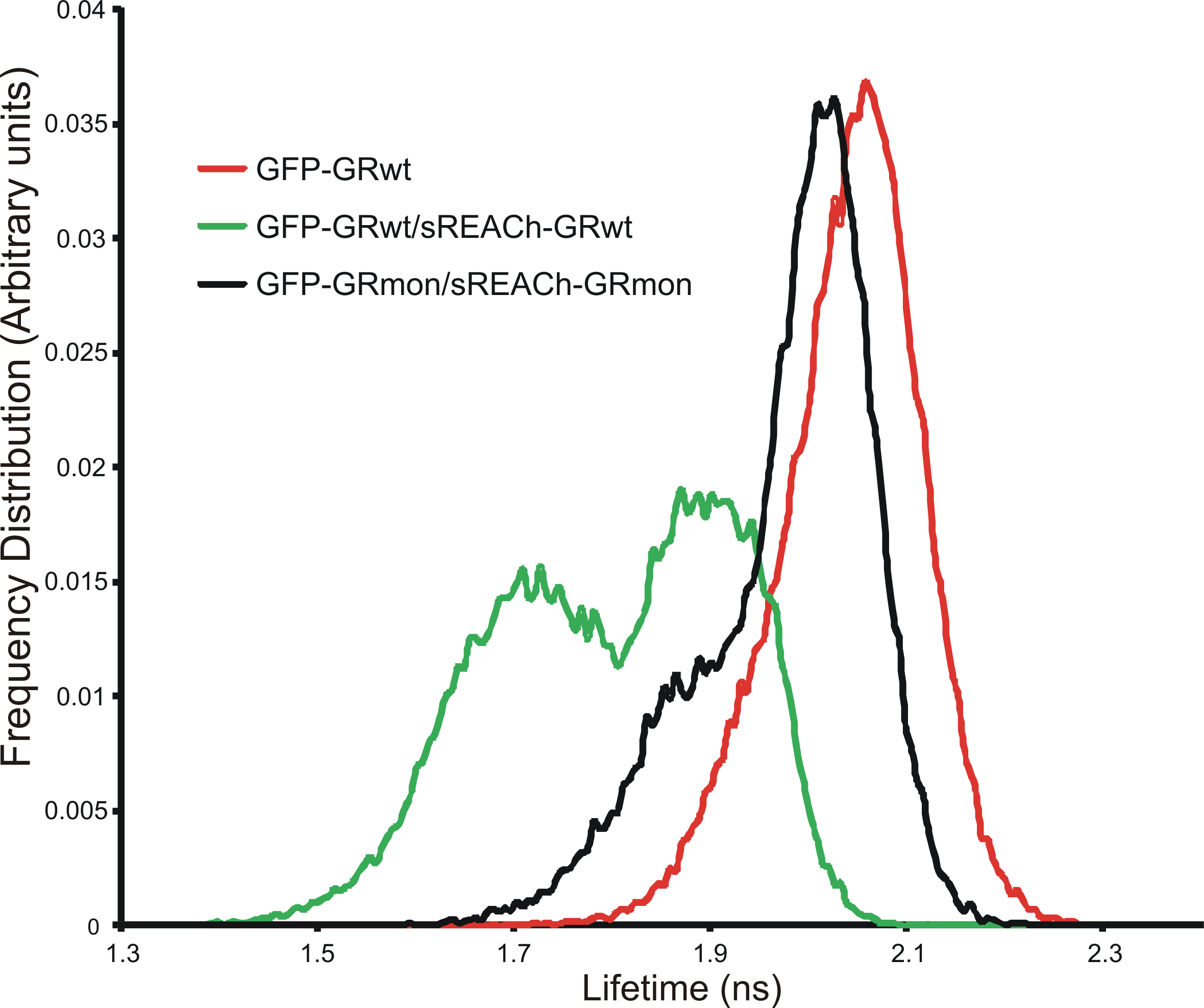

Supplement: Figure S3 — FLIM-FRET analysis on GRwt and GRmon. 3617 cells were transiently transfected with the indicated combination of plasmids and treated for 30 min with 100 nM Cort. Frequency distribution for the eGFP photon lifetimes with different donor-acceptor combinations (n = 25–26 cells per condition) is shown. The constructs are identical aside from the indicated mutations in GR and the fluorophore tag. eGFP-GRwt/sREACh-GRwt shifts to reduced lifetimes values relative to the control (no FRET) indicating interaction of the alternatively tagged GRwt proteins. In contrast, the higher lifetime values for eGFP-GRmon/sREACh-GRmon indicate impaired interaction examined by FRET. The binomial distribution of lifetimes in the eGFP-GRwt/sREACh-GRwt pairing could be due to different FRET efficiencies produced by varied transient transfection of donor to acceptor across the cell population. (TIF) [file pbio.1001813.s003.tif]

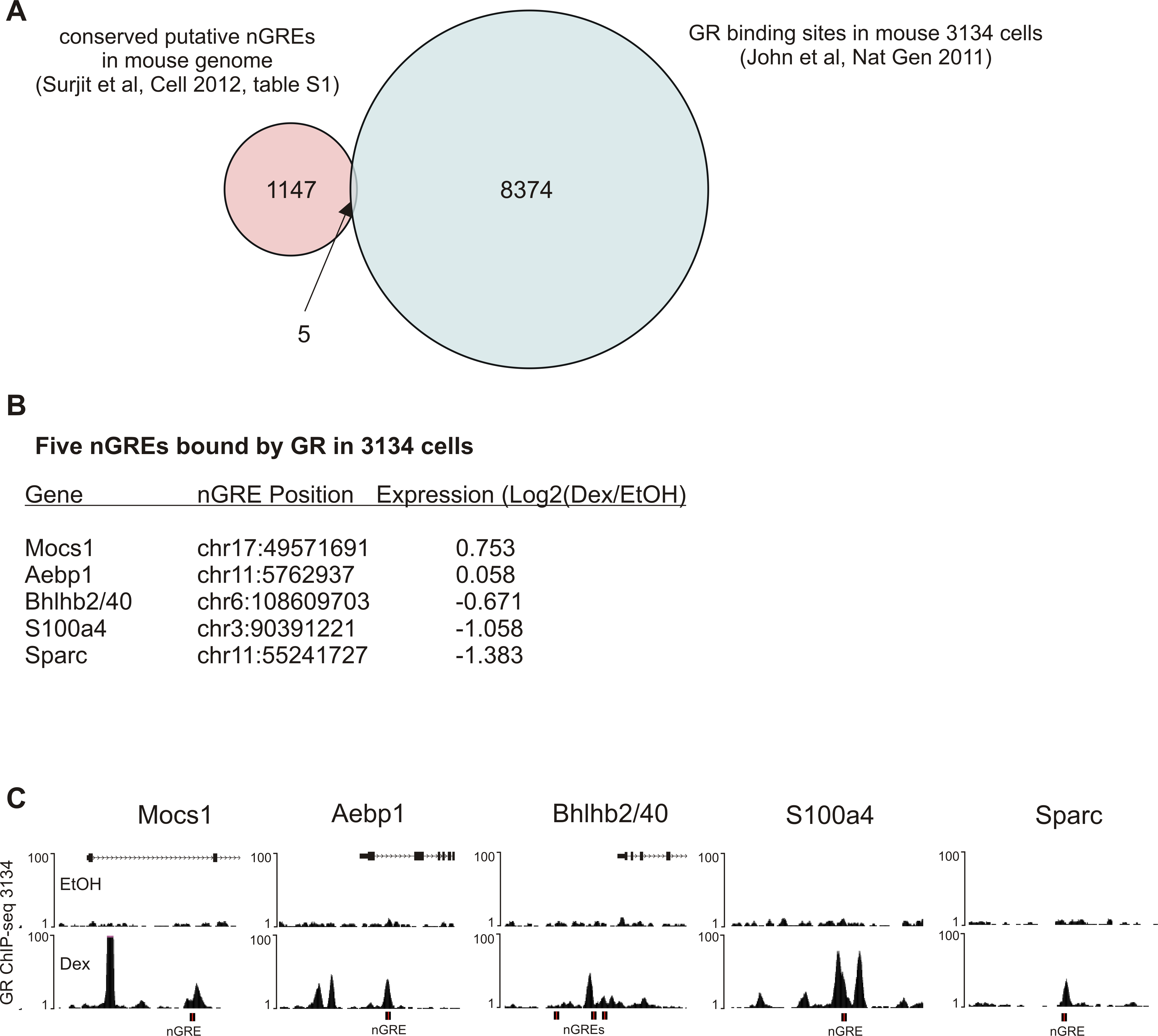

Supplement: Figure S5 — Searching for nGRE in 3134 cells. (A) Venn diagram generated by overlapping all putative nGREs conserved between human and mouse (taken from Surjit et al., Table S1 [34]) with GR ChIP-seq data (taken from John et al. [33]). (B) The table shows the genome localization (mm9) of each nGRE found (named after the nearby gene). We also show the relative expression according to previously published microarray data [35]. (C) Genome browser shots of the nGREs from GR ChIP-seq data [33]. (TIF) [file pbio.1001813.s005.tif]
